# First Detection of *Acinetobacter baumannii* in *Pediculus humanus capitis* from Latin America

**DOI:** 10.3390/tropicalmed8070345

**Published:** 2023-06-29

**Authors:** Kelsey Larkin, Ariel Ceferino Toloza, Jose Antonio Gabrie, Carol A. Rodríguez, Maria Mercedes Rueda, Gabriela Matamoros, Oscar Palacio, Shabana Jamani, Gustavo Fontecha, Ana L. Sanchez

**Affiliations:** 1 Department of Health Sciences, Faculty of Applied Health Sciences, Brock University, 1812 Sir Isaac Brock Way, St. Catharines, ON L2S 3A1, Canada; kl15ly@brocku.ca (K.L.); jgabrie@brocku.ca (J.A.G.); op15kb@brocku.ca (O.P.); sj13rg@brocku.ca (S.J.); 2 Centro de Investigaciones de Plagas e Insecticidas (CONICET-UNIDEF), Villa Martelli, Buenos Aires 1603, Argentina; atoloza@conicet.gov.ar; 3 Department of Parasitology, Universidad Nacional Autónoma de Honduras, Boulevard Suyapa, Tegucigalpa 11101, Honduras; carol.rodriguez@unah.edu.hn (C.A.R.); maria.rueda@unah.edu.hn (M.M.R.); 4 Microbiology Research Institute, Universidad Nacional Autónoma de Honduras, Boulevard Suyapa, Tegucigalpa 11101, Honduras; gabriela.matamoros@unah.edu.hn (G.M.); gustavo.fontecha@unah.edu.hn (G.F.)

**Keywords:** *Pediculus humanus capitis*, *Acinetobacter baumannii*, head lice, Argentina, Colombia, Honduras, Latin America

## Abstract

Several studies have documented the presence of *Acinetobacter baumannii,* a known multi-drug-resistant pathogen, in the human head louse, *Pediculus humanus capitis.* Since no reports from countries in Latin America have been published, the aim of the present study was to determine whether *A. baumannii* was present in head lice specimens collected in this geographic region. Head lice specimens from Argentina, Colombia, and Honduras were analyzed. PCR assays were performed to confirm the specimens’ species and to investigate whether the DNA of *A. baumannii* was present. The products of the latter were sequenced to confirm bacterial identity. Altogether, 122 pools of head lice were analyzed, of which two (1.64%) were positive for *A. baumannii*’s DNA. The positive head lice had been collected at the poorest study site in Honduras. The remaining specimens were negative. This study is the first to report the presence of *A. baumannii* in human head lice from Latin America. Further investigations are required to elucidate whether these ectoparasites can serve as natural reservoirs or even effectively transmit *A. baumannii* to humans.

## 1. Introduction

The human head louse, *Pediculus humanus capitis* (De Geer, 1767), is an obligate blood-feeding wingless insect (Anoplura: Pediculidae) that completes its lifecycle on the scalp, causing an infestation termed pediculosis capitis [1,2]. Head lice are transmitted by direct head-to-head contact and are most prevalent in children, causing scalp itching, irritability, and sleep disturbance. The infestation may also foster secondary bacterial infections as a result of the excoriations produced by scratching [3]. Head lice transmission can also occur in other settings, such as health care centres and hospitals, especially children’s outpatient units. Studies conducted on Brazilian children attending public hospitals reported an infestation prevalence of 2.8% [4] and 6.1% [5]. Although unusual, health-care-associated transmission of pediculosis is possible due to the close contact between staff and infested pediatric patients [6]. Several reports demonstrate that head lice transmission in medical settings is highly likely [4,5,7,8].

Head lice are not considered vectors of human infectious agents, as is the case with the other ecotype of the species, *Pediculus humanus humanus*, commonly known as the body louse. Body lice are known to transmit *Borrelia recurrentis*, *Borrelia quintana*, and *Rickettsia prowasekii* [9,10,11]. In addition, a recent review suggests the possibility that the body louse may transmit *Yersinia pestis*, the etiologic agent of the plague [12]. Another human pathogen found in body louse is *Acinetobacter baumannii*, as shown by La Scola and Raoult, who found that 21% of collected specimens were naturally infected with this bacterial species [13]. Using an experimental model, Houhamdi and Raoult demonstrated that the human body louse was not only susceptible to being infected by *A. baumannii* but was also able to maintain the infection (both mouse- and human-strain) [14].

In terms of head lice, evidence produced in the last decade indicates that they can harbor human pathogens, even though it is believed that their stronger immune response—as compared to that of *P. h. humanus*—hinders bacterial proliferation, making them unsuitable vectors [15]. Bacterial pathogens identified from head lice include several species of *Acinetobacter* (e.g., *A. baumannii*, *A. johnsonii*, and *A. variabilis*), as well as *Coxiella burnetii*, *Bartonella quintana*, *B. recurrentis*, *Borrelia theileri*, *Y. pestis*, *Rickettsia aeschlimannii,* and *Serratia marcescens* [9,16,17,18,19]. In laboratory experiments, head lice have been able to transmit *R. prowazekii* but have yet to be reported as vectors during outbreaks of louse-borne epidemic typhus, although arguments are made that head lice have been overlooked during outbreak investigations [20]. For these reasons, head lice vectorial competence is still under examination [12].

Among these pathogenic bacteria, special attention has been given to *A. baumannii*, a Gram-negative bacterium of widespread distribution known as an opportunistic organism causing frequent healthcare-associated outbreaks [21]. This bacterium is highly resistant to environmental conditions and, due to its capacity for biofilm formation, can survive on dry surfaces for long periods of time [22]. In addition, *A. baumannii* isolates are well documented as multi-drug and extensively drug-resistant bacteria [22]. Carbapenem-resistant *A. baumannii* infections in critically ill patients often lead to serious health outcomes such as pneumonia, sepsis, and meningitis, as well as urinary and wound infections [23]. *A. baumannii* belongs to the ESKAPE pathogens (also including *Enterococcus faecium, Staphylococcus aureus, Klebsiella pneumoniae, Pseudomonas aeruginosa,* and *Enterobacter cloacae*), responsible for the majority of nosocomial infections [24].

Since head lice persist almost everywhere as the most common human ectoparasite [25], bacterial detection in *P. humanus capitis*, especially that of *A. baumannii,* raises concerns and requires more investigation [9,16]. It is necessary to confirm whether this is a worldwide occurrence as well as define what role, if any, head lice are playing in the transmission of these bacteria to humans [19].

Given that studies on pathogenic bacteria in human head lice have not been reported in Latin America, the aim of the present work was to determine whether *A. baumannii* was present in head lice specimens collected in three Latin American countries from which previous studies on childhood infestations have demonstrated that this ectoparasitosis is of public health significance. In a small community in rural Honduras (one of the study sites for the present investigation), the proportion of head lice infestation in children 2–14 years of age was 83% [26], while a national survey of >15,000 children in 2014 found a pediculosis capitis prevalence of 9.9% [27]. In Argentina, a study in Buenos Aires in 2018 reported prevalence figures ranging from 26.4% to 69% in children enrolled in several primary schools [28]. In Colombia, Medina–Ortega et al. reported that the prevalence and incidence of pediculosis capitis among 356 children in a low-income site were 5.1% and 20.2%, respectively [29]. In a study in a daycare in urban Bogota, the prevalence of pediculosis among 178 children ranged from 2.12% to 18.7% in a 7-month period [30]. As in many countries, pediculosis in Argentina, Colombia, and Honduras is a neglected infestation not prioritized in national or regional public health programs [31].

## 2. Materials and Methods

### 2.1. Insect Specimens

*Pediculus humanus capitis* specimens were collected from schoolchildren from three different countries: Argentina, Colombia, and Honduras.

Argentina: specimens from Ciudad Autónoma de Buenos Aires, Argentina (34°36′13.26″ S 58°22′53.61″ W), were collected in 2017 from school children attending several urban schools. Lice were collected from children’s dry hair with the aid of a fine-tooth comb and preserved in 70% ethanol for further analysis.

Colombia: specimens were collected in 2018 from children attending “Centro Solidaridad en Marcha Juan Pablo II” located in an urban area of Medellín, Department of Antioquia, Colombia (6°15′ N 75°37′ W). Lice were collected from children’s dry hair with the aid of a V-Comb, as reported by Jamani et al. [26], and preserved in 70% ethanol for further analysis.

Honduras: specimens were collected from school children in two rural sites: (i) in La Hicaca, department of Yoro, in 2016 during Jamanis’ et al.’s qualitative study [26], and (ii) in the municipality of San Buenaventura, department of Francisco Morazán, in 2019, during a community service visit. Lice were collected from children’s dry hair with the aid of a V-Comb and preserved in 70% ethanol for further analysis. The geographic coordinates for these two sites are: La Hicaca, 15°27′ N, 87° 2′ W; and San Buenaventura, 13°54′ N 87°12′ W.

In all, a total of 122 vials containing insects were obtained for analysis: 50 from Argentina, 25 from Colombia, 40 from La Hicaca, Honduras, and 7 from San Buenaventura, Honduras. Each vial contained a variable number of lice, but altogether, 368 insects were available for analysis. Specimens were kept in 1.5 mL Eppendorf tubes containing a 70% ethanol solution at 4 °C until analysis.

### 2.2. Identification, Separation, and Dissection of Lice Specimens

Lice were separated from hair, debris, molt, and nits. The sex and life stage of the lice were identified under a stereoscope (Ken-A-Vision™, Ken-A-Vision™ Vision Scope 2 Stereo Microscope (Ken-A-Vision Mfg. Co., Inc. Kansas City, MI, USA) at 20× magnification. Identification keys for morphological characteristics described by Al-Marjan et al. [32] were followed to ensure the insects’ species.

From each of the original 122 vials, between 1 and 5 lice were selected, depending on their size, placed in sterile vials, washed twice with sterile deionized water, and then, using aseptic techniques, cut longitudinally by hand using a sterile stainless-steel blade #22 scalpel in order to create a corresponding number of vials in duplicate, which were kept at −20 °C. One set was used for the present study, while the other was saved for future studies.

### 2.3. Detection of Acinetobacter baumannii DNA in Head Lice

Prior to processing lice for extraction of bacterial DNA and PCR analysis, a positive bacterial DNA control was prepared from a pure culture of *A. baumannii* (ATCC 19606, Cedarlane Labs, Burlington, ON, Canada).

### 2.4. DNA Extraction

DNA from *P. h. capitis* and *A. baumannii* were both extracted using QIAmp DNA mini kits from Qiagen (Hilden, Germany), following the manufacturer’s instructions. Modifications for lice included (a) the addition of three 2-mm stainless steel beads before incubation to facilitate breakage of the exoskeleton; (b) during the last step of extraction, 100 μL of buffer AE was added instead of 200 μL.

### 2.5. Polymerase Chain Reaction

PCR assays were used to verify the DNA of *P. humanus* and to investigate the potential presence of DNA of *A. baumannii* within lice samples. Methods described by Raoult and collaborators [33], as well as Azad et al. and Fournier et al. were followed [34,35].

First, *P. humanus* DNA was confirmed through PCR. Reactions contained 12.5 μL of Taq Master Mix 2× (Promega, Madison, WI, USA), and 1.5 μL of each primer (10 μM) targeting the NADH dehydrogenase subunit (NAD4) gene. (Forward: TTG TTG TGC TTT TGA CTT CTT G, reverse: CCC TGA TTT GAA GTA TTA AAG AAA CTC), 1 μL of DNA (40 ng/μL), and nuclease-free water for a total volume of 25 μL [33]. The PCR program was as follows: 1 cycle at 95 °C for 3 min; 44 cycles of 95 °C for 30 s, 58 °C for 30 s, and 72 °C for 90 s; and a final extension step at 72 °C for 7 min.

For *A. baumannii* identification, the PCR reaction contained 25 μL of Taq Master Mix 2× (Promega, Madison, WI, USA), 2 μL of each primer (10 μM)—targeting the *rec*A gene for recombinase gene (Abau2F: 5′-ACA TCG CAT TGG GGA TTG GT-3′, Abau2R: 5′-GAC GCG CTT GTA GAC CCA TA-3′), 18.0 μL of NFW, 1.0 μL of bovine serum albumin acetylated (Promega, Madison, WI, USA), and 2 μL of DNA (40 ng/μL). The PCR reaction was programmed as follows: 94 °C for 5 min; 35 cycles of 94 °C for 60 s, 60 °C for 60 s, and 72 °C for 60 s; and a final extension step at 72 °C for 10 min. Negative controls consisted of PCR mixtures or water.

PCR products of 366 bp were separated by electrophoresis on 2% agarose gels with ethidium bromide.

The amplification products were sequenced on both strands using the same primers used for amplification. Sequencing services were provided by Psomagen^®^ (www.psomagen.com, Rockville, MD, USA; Accessed on 2 June 2023). The sequences were trimmed and edited with the Geneious^®^ 9.1.7 software and deposited into the NCBI GenBank. The obtained sequences were analyzed with the GenBank BLAST tool to confirm their identity as *A*. *baumannii.*

## 3. Results

Two vials out of the 122 analyzed (1.64%) tested positive for *A. baumannii’s* DNA. The positive head lice had been collected in La Hicaca, Honduras. The remaining specimens from Argentina, Colombia, and San Buenaventura, Honduras, were negative for *A. baumannii.*

**Figure 1:**
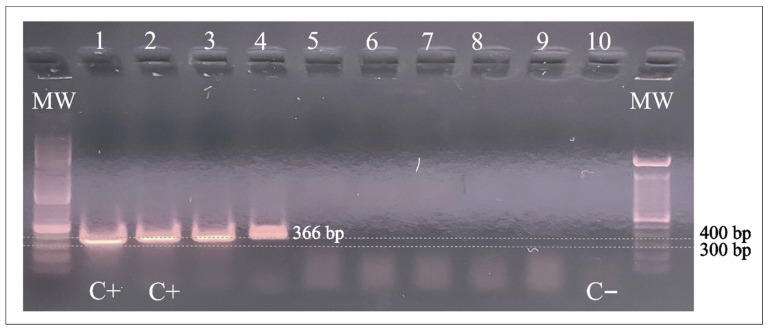
PCR amplification of *A. baumannii* DNA in head lice specimens from Honduras. Lanes 1 and 2: *A baumannii* positive control; Lanes 3 and 4: positive samples; Lanes 5–9: negative samples. Lane 10: Negative control. C+: positive control; C−: negative control; MW: molecular weight; bp: base pairs.

Figure 1 shows the PCR (366 bp) products of the two pooled samples containing halves of three different lice each. These positive specimens were collected in Honduras from children living at the poorest study site.

The sequences obtained here were confirmed as a partial sequence of the *rec*A gene of *A. baumannii* with 100% identity and query coverage of 100%. All the sequences obtained turned out to be identical, with a size of 306 nucleotides and a sequence of 102 amino acids when translated in silico. One sequence was deposited in GenBank under the accession number ON564437.

## 4. Discussion

This is the first report on the positive identification of *A. baumannii* infecting head lice from any country in Latin America. Out of the three countries investigated, only lice obtained in Honduras were positive for *A. baumannii’s* DNA.

In the countries where lice were collected, reports of *A. baumannii* infections are almost exclusively healthcare-associated. For example, in Honduras, a cross-sectional study conducted at a tertiary health care centre reported that the majority of isolates were from patients in the intensive care unit (ICU), with a significant proportion (44%) hospitalized in the pediatric wards (neonatal ICU and general pediatrics) [36]. In Argentina, carbapenem resistance in *A. baumannii* has been monitored since the year 2000, and Argentina is the Latin American country with the most scientific reports on this infection [37]. In Colombia, a recent 3-year study found that *A. baumannii* comprised 20% of the isolates of the World Health Organization’s Gram-negative priority bacteria [38]. Overall, Latin America has been identified as a geographical area where carbapenem-resistant *A. baumannii* is an emerging health problem [39]. Investigating whether *A. baumannii* could be found associated with head lice is an interesting approach to ascertaining sources of this pathogen’s carriage at the community level.

Studies from African and European countries, as well as from the U.S., have also confirmed the presence of *A. baumannii* and other bacterial species in head lice, with frequencies ranging from 3.6% to 81% [40]. Head lice collected from school children in Thailand and France found a low prevalence of *A. baumannii,* i.e., 1.45% and 2.7%, respectively [41,42]. Similarly, lice from children in welfare homes in Malaysia, southeastern Asia, found that 24.2% of the lice examined contained DNA of *Acinetobacter* spp. and 5.8% contained *A. baumannii* specifically [9]. A higher prevalence has been reported in the Democratic Republic of the Congo by Koyo et al. These authors detected six *Acinetobacter* species, including *A. baumannii,* in 8.3% of the samples. They also identified a new potential species named *Candidatus Acinetobacter pediculi* [18]. A prevalence of over 10% has been reported in France (33%) [16] and Algeria (27%) [19], while the highest prevalence reported in the literature at this time is the work by Kempf et al., who examined a total of 115 head lice from healthy individuals in Ethiopia and found a staggering 67% positivity for *A. baumannii* [17].

It appears that isolating *A. baumannii* in head lice is associated with poverty and a lack of sanitation [11]. Conversely, most studies on head lice-infesting populations living under adequate hygienic conditions have failed to detect these pathogenic bacteria [12]. The findings of the present study support these observations, as *A. baumannii* was only detected in lice from children residing in the study site of the lowest socio-economic status (La Hicaca, Honduras). This finding, along with those of Zuniga-Moya et al. that most cases of this pathogen in Honduras were isolated from children in the pediatric wards [36], suggests that careful inspection and mitigation measures for head lice in hospitalized children could provide benefits beyond ectoparasite control.

The coexistence of *A. baumannii* within head lice raises important questions, the first of which is whether the strain of *A. baumannii* found in head lice can effectively produce human infection. If this were the case, it would be necessary to determine whether head lice could in fact naturally transmit—either mechanically or biologically—this bacterial pathogen (or at least whether these ectoparasites play a significant role as reservoirs for *A. baumannii* in nature). It would also be important to elucidate how head lice usually acquire *A. baumannii* from their human host, either from the host’s blood in cases of bacteremia or from the skin microbiome.

One of the main limitations of this study is that we did not try to culture bacteria from the lice, as in order to preserve DNA, insects were deposited in alcohol. Similarly, no attempts were made to obtain scalp swabs from infested children to further understand the origin of the bacterial DNA within the lice. Lastly, molecular differentiation between body and head lice was not attempted [43]. However, we are confident that the studied specimens are indeed the head lice ecotype, as all lice were collected exclusively from the heads of infested children. Furthermore, there is no history of body lice infestations in the schools where the insects were collected.

Despite these limitations, the present work generated good-quality results, which contributed to expanding the geographic areas reporting the association between head lice and *A. baumannii.*

While data on *A. baumannii* transmission in Honduras, Argentina, and Colombia are still limited to health-care-associated infections, evidence of this bacterial species isolation from sources such as the environment, human body, and head lice is growing. Due to the ubiquitous presence of *A. baumannii* in nature, community-acquired infections as well as human community carriage must be more common than presently reported [44]. The present work, as well as previous investigations, emphasize the need for intensified research efforts to determine whether head lice can serve as important community reservoirs and/or potential carriers of this pathogen to humans. Importantly, this work and others’ support a paradigm shift with regards to the clinical importance of head lice and may help foster efforts for their prevention and control.

## Data Availability

Not applicable.
